# Intergenerational Service-Learning During COVID-19

**DOI:** 10.1093/geroni/igab046.1556

**Published:** 2021-12-17

**Authors:** Tamar Shovali

**Affiliations:** Eckerd College, Eckerd College, Florida, United States

## Abstract

Mentor Up is a technology training program designed to reduce loneliness through technology training and intergenerational relationships. The program, which has similarities to Cyber Seniors, has been held at Eckerd College for four years and has traditionally been held in-person at a local Continuing Care Retirement Community (CCRC). During the pandemic we partnered with AARP to re-think the program and offer a modified version of Mentor Up on Zoom. Five one-hour one-on-one virtual technology training sessions led by 16 students were scheduled. Participants joined to ask questions about how to use features on their smartphones or how to navigate the Zoom virtual platform more effectively. The remote nature of programming allows for expanded 2021 participation, including AARP members across Florida and all three Westminster Communities of Florida in the area (CCRC, ALF, SNF). Participation rates, strategies to implement intergenerational programs on a virtual platform, and lessons learned will be highlighted.

